# Manzamine A Exerts Anticancer Activity against Human Colorectal Cancer Cells

**DOI:** 10.3390/md16080252

**Published:** 2018-07-29

**Authors:** Li-Chun Lin, Tzu-Ting Kuo, Hsin-Yi Chang, Wen-Shan Liu, Shih-Min Hsia, Tsui-Chin Huang

**Affiliations:** 1 Ph.D. Program for Cancer Molecular Biology and Drug Discovery, College of Medical Science and Technology, Taipei Medical University and Academia Sinica, Taipei 11031, Taiwan; lisa81318@gmail.com; 2 Graduate Institute of Cancer Molecular Biology and Drug Discovery, College of Medical Science and Technology, Taipei Medical University, Taipei 11031, Taiwan; taros71526@hotmail.com (T.-T.K.); cindy20028@yahoo.com.tw (W.-S.L.); 3 Graduate School of Pharmaceutical Sciences, Kyoto University, Kyoto 6068501, Japan; hsinyi.chang@pharm.kyoto-u.ac.jp; 4 School of Nutrition and Health Sciences, College of Nutrition, Taipei Medical University, Taipei 11031, Taiwan; bryanhsia@tmu.edu.tw; 5 Graduate Institute of Metabolism and Obesity Sciences, College of Nutrition, Taipei Medical University, Taipei 11031, Taiwan; 6 School of Food Safety, College of Nutrition, Taipei Medical University, Taipei 11031, Taiwan; 7 TMU Research Center of Cancer Translational Medicine, Taipei Medical University, Taipei 11031, Taiwan

**Keywords:** manzamine A, cell cycle, apoptosis, epithelial–mesenchymal transition, colorectal cancer

## Abstract

Marine sponges are known to produce numerous bioactive secondary metabolites as defense strategies to avoid predation. Manzamine A is a sponge-derived β-carboline-fused pentacyclic alkaloid with various bioactivities, including recently reported anticancer activity on pancreatic cancer. However, its cytotoxicity and mode of action against other tumors remain unclear. In this study, we exhibit that manzamine A reduced cell proliferation in several colorectal cancer (CRC) cell lines. To further investigate the manzamine A triggered molecular regulation, we analyzed the gene expression with microarray and revealed that pathways including cell cycle, DNA repair, mRNA metabolism, and apoptosis were dysregulated. We verified that manzamine A induced cell cycle arrest at G_0_/G_1_ phase via inhibition of cyclin-dependent kinases by p53/p21/p27 and triggered a caspase-dependent apoptotic cell death through mitochondrial membrane potential depletion. Additionally, we performed bioinformatics analysis and demonstrated that manzamine A abolished epithelial–mesenchymal transition process. Several mesenchymal transcriptional factors, such as Snail, Slug, and Twist were suppressed and epithelial marker E-cadherin was induced simultaneously in HCT116 cells by manzamine A, leading to the epithelial-like phenotype and suppression of migration. These findings suggest that manzamine A may serve as a starting point for the development of an anticancer drug for the treatment of metastatic CRC.

## 1. Introduction

Colorectal cancer (CRC) is the third-most common cancer in both genders and the fourth leading cause of cancer related mortality, responsible for 9.7% of cancer-related deaths worldwide [1,2,3]. Distant metastasis caused by disease recurrence and development of drug resistance is the main cause of death in CRC patients [4,5]. In 2017, an estimated 135,430 new cases will be diagnosed with colorectal cancer and about 50,260 people will die from this disease in the US [6].

Accumulated evidences of genetic mutations and epigenetic instability on oncogene activation and tumor suppressor inactivation have been reported to be central molecular and pathophysiological events to initiate CRC [7,8]. The most frequent aberrations found in CRC patients are mutations in adenomatous polyposis coli (APC), catenin-β1 (CTNNB1), family with sequence similarity 123B (FAM123B; also known as AMER1), kirsten rat sarcoma viral oncogene homolog (KRAS), B-Raf proto-oncogene, serine/threonine kinase (BRAF), erb-b2 receptor tyrosine kinase 2 E (RBB2), SMAD family member 4 (SMAD4), phosphatidylinositol-4,5-bisphosphate 3-kinase catalytic subunit-α (PIK3CA), transforming growth factor-β receptor 2 (TGFBR2), AT-rich interactive domain 1A (ARID1A), and tumor Protein P53 (TP53). These mutations promote tumorigenesis by perturbing the key signaling pathways, such as WNT–β-catenin, epidermal growth factor (EGF)–mitogen-activated protein kinase (MAPK), phosphatidylinositol 3-kinase (PI3K)–AKT, and TGFβ signaling pathways, or affecting genes that regulate fundamental processes, such as DNA repair, cell cycle progression and proliferation [2,9].

Several therapeutic strategies are developed to overcome CRC. However, server side effects due to drug toxicity towards normal tissues and development of drug resistance are occurred in CRC patients receiving conventional chemotherapy. Therefore, development of novel anticancer therapeutic alternatives for CRC is still urgently required. Manzamine A (Manz A), comprising a pentacyclic core coupled to a β-carboline alkaloid, is isolated from sponges of the genera *Haliclona* sp., *Xestospongia* sp. and *Pellina* sp. [10,11,12]. Manz A was first isolated from *Haliclona* sp. in Okinawa sea and reported to display anticancer activity against leukemia cells [13]. It possesses a diverse range of potent bioactivities including insecticidal, antibacterial, and antileishmaniasis effects, and anti-malarial, anti-inflammatory, and antiviral activities [10,14,15,16,17,18,19]. A previous study demonstrated that Manz A decreased single cell formation, abrogated cell migration, and sensitized AsPC-1 pancreatic adenocarcinoma cells towards TRAIL induced apoptosis [20]. A recent work also reported that Manz A targeted vacuolar ATPases and inhibited autophagy in pancreatic cancer cells, suggesting a promising strategy for the treatment of cancer [21]. Nevertheless, the effects of Manz A on CRC and their mechanisms remain unclear. In the present study, we attempted to investigate the anti-tumor properties of Manz A in HCT116 human colorectal carcinoma cells. We showed that Manz A significantly inhibited the proliferation of several CRC cell lines. By combining enrichment analysis and network analysis on microarray data, we found that Manz A reduced the expression of genes involved in several fundamental pathways and activated the apoptotic gene expression. The effects of MA on cell cycle progression, apoptosis, epithelial–mesenchymal transition (EMT) process, and cell migration were further validated.

## 2. Results

### 2.1. Manz A Inhibits Cell Proliferation in Human Colorectal Carcinoma Cells

**Figure 1:**
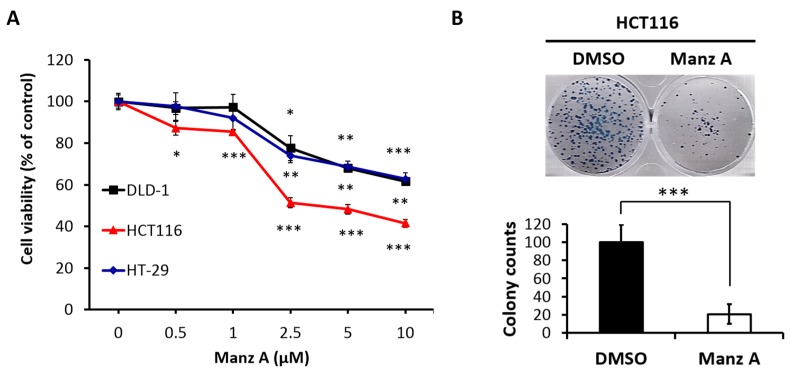
Manz A reduced cell proliferation in colorectal cancer cells. (**A**) HCT116, DLD-1, and HT-29 cells were treated with Manz A at various concentrations of 0, 0.5, 1, 2.5, and 5 μM for 24 h. Cell viability (%) was measured using MTS cell proliferation assay and data was expressed as percentage of absorbance from Manz A treated cells compared to DMSO treated ones; (**B**) Colony formation assay was performed to determine the long-term effects of Manz A on the growth of HCT116 cells. Cells were pre-treated with 0.1% DMSO or 5 μM Manz A for 24 h and left for 7 days to grow. Colonies were then stained with Giemsa. The data were expressed as the mean ± SD of three independent experiments. * *p* < 0.05, ** *p* < 0.01, *** *p* < 0.001.

To evaluate the effects of Manz A on the proliferation of human colorectal carcinoma cells, we performed the MTS assay on HCT116, HT-29, and DLD-1 cells in a dose-dependent manner at concentrations of 0, 0.5, 1, 2.5, 5, and 10 μM for 24 h. We found that Manz A significantly decreased the cell viability of all colorectal carcinoma cells and showed a higher efficacy on HCT116 compared with HT-29 and DLD-1 (Figure 1A). The IC50 values were 4.5 ± 1.7 μM in HCT116 cells and more than 10 μM in DLD-1 and HT-29. To determine the long-term inhibitory effect of Manz A on the proliferation of HCT116 cells, we performed a colony formation assay. The 24-h treated cells were seeded in 6-well plates and cultured in drug-free medium for one week. We showed that Manz A significantly reduced the number and size of colonies without continued exposure to the drug (Figure 1B), suggesting that Manz A caused an irreversible cell proliferation inhibition.

### 2.2. Manz A Reduces Gene Expressions Involved in Several Fundamental Pathways

**Figure 2:**
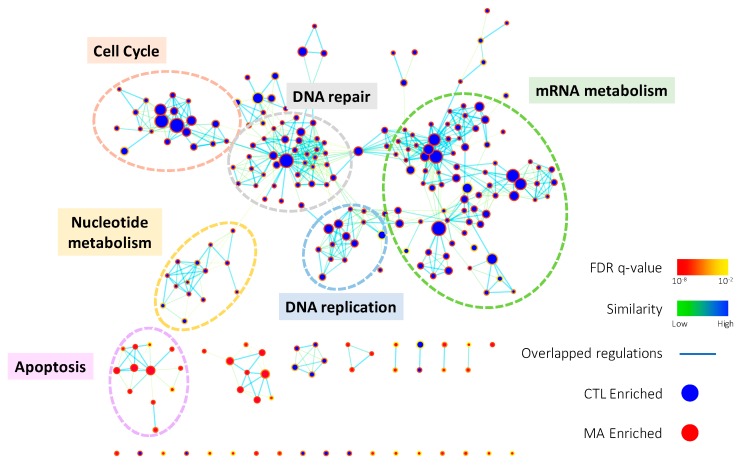
The enrichment map of gene ontology biological processes (GOBPs) enriched in GSEA result. GSEA was performed on microarray data to enrich terms in GOBP. Enrichment results were visualized with Cytoscape 3.0 using Enrichment Map plugin. Each node indicates an enriched term and edges represent overlapped genes between terms. The color of node boarder and edge were shown according to enrichment FDR and similarity, respectively. Blue and red nodes refer to enriched functions in DMSO vehicle control (CTL) and Manz A treatment. The size of node and edge width represents the number of genes enriched in each term and overlapped between terms, respectively.

To comprehensively elucidate the regulation of Manz A on HCT116 cells, we profiled the expression of ~40,000 genes by microarray analysis. A total of 1574 genes were significantly regulated (*p* < 0.05) and 639 of them were more than 2-fold changes (FC) with 308 upregulated and 311 downregulated transcripts. To find out the connection between gene expression and biological function, we used two enrichment analyses, over-representation analysis (ORA), and functional class sorting (FCS), against pre-defined sets of gene lists. In ORA, we revealed that Manz A caused the downregulation in several pathways including cell cycle, DNA repair, mRNA metabolism, mitochondrial electron transport chain (ETC), and transcription (Figure S1). For FCS, we applied gene set enrichment analysis (GSEA) and integrated the influences of Manz A in biological process and activity of transcriptional regulation. With the consistency with ORA result, most downregulated gene ontology terms of biological process (GOBP) were belonged to cell cycle, DNA replication, mRNA metabolism, and DNA repair, while one upregulated GOBP term related to apoptosis was enriched (Figure 2). In transcriptional activity analysis, we found that E2F Transcription Factor 1 (E2F) was the most downregulated transcription factor, while cAMP response element binding protein (CREB), activating transcription factor (ATF) and activator protein 1 (AP1) were upregulated in transcriptional activity (Figure S2). Together, we suggest that Manz A inhibition of cell proliferation might be due to the diminishment in cell cycle progression and the initiation of apoptotic cell death.

### 2.3. Manz A Induces Cell Cycle Arrest at G_0_/G_1_ Phase

**Figure 3:**
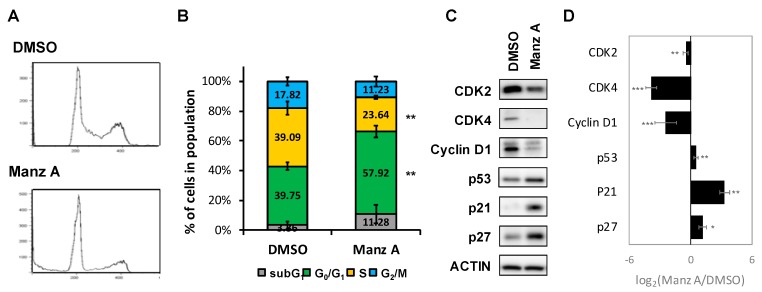
Manz A induced G_0_/G_1_ phase arrest. (**A**) HCT116 cells were treated with 5 μM Manz A for 24 h and then subjected to DNA content analysis by flow cytometry. The cell cycle distribution was quantified with model fitting in FlowJo; (**B**) Cell cycle distributions from three independent experiments is shown. * *p* < 0.05, ** *p* < 0.01, *** *p* < 0.001; (**C**) Representative western blot analyses of the cell cycle regulator levels in response to Manz A treatment in HCT116 cells. ACTIN was used as an internal control; (**D**) Relative expression level of each protein was densitometrically estimated and normalized to that of ACTIN under the same treatment. Data is presented in log_2_ ratio of the protein level in Manz A treated cells to that in DMSO treated ones from three independent results.

According to the findings from microarray analysis, we designed a series of experiments to validate the mode of cell death induced by Manz A. First, we performed flow cytometry analysis to measure the changes in cell population distribution whinin cell cycle. We revealed that Manz A increased the cell population at G_0_/G_1_ phase by 1.46 fold and decreased that at S phase by 1.65 fold simultaneously (Figure 3A,B), indicating that Manz A markedly induced cell cycle arrest at G_0_/G_1_ phase.

Aberrant cell division and uncontrolled proliferation are often associated with deregulation of cell cycle kinases in cancer cells. Cell cycle progression is regulated by several cyclin-dependent kinases (CDKs) that act in complex with cyclins. Cyclin Ds–CDK4/6 complexes drive cell cycle progression from G_0_ or G_1_ phase into S phase, in which DNA replication occurs. The complex sequester CDK inhibitors (CKIs), p21^CIP1^ and p27^KIP1^, bind to and prevent activation of cyclin E–CDK2 kinase, driving cell cycle progression from G_0_ or G_1_ phase into S phase. While DNA damage occurs, p21 is activated by p53 to arrest cell cycle progression [22]. To determine the effect of Manz A on protein expression of cell cycle regulators, we conducted western blot analysis and found that Manz A obviously decreased the protein expression of CDK2, CDK4 and cyclin D_1_ and increased levels of CKIs including p21^CIP1^, p27^KIP1^, and p53 (Figure 3C). We imply that cell cycle arrest was involved in Manz-A-inhibited cell proliferation.

### 2.4. Manz A Induces Caspase-Dependent Apoptotic Cell Death

**Figure 4:**
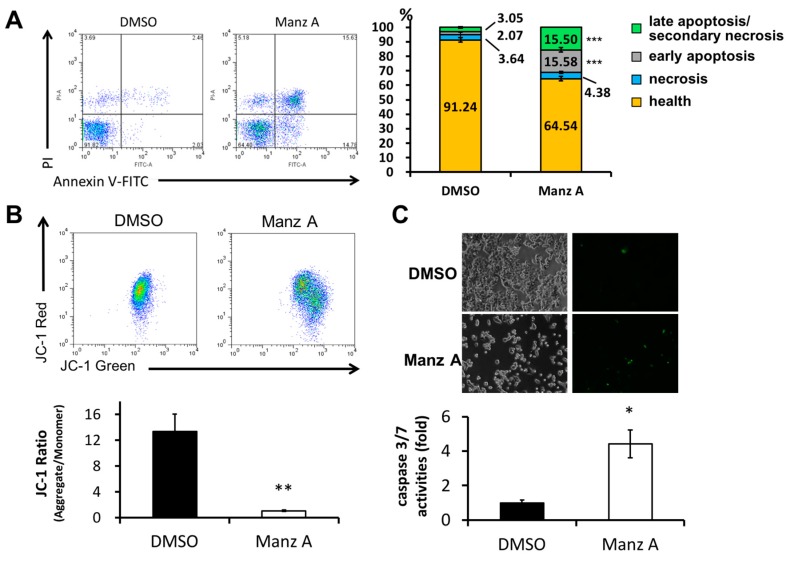
Manz A induced apoptosis in HCT116 cells. Cells were treated with 5 µM Manz A for 24 h. (**A**) Cells were harvested and stained with Annexin V-FITC and propidium iodide (PI). The fluorescent signal was measured by flow cytometry. A representative result is shown in the left panel and statistics analysis is at the right panel; (**B**) Cells were subjected to mitochondrial membrane potential detection by JC-1 and flow cytometry; (**C**) Cells were subjected to caspase 3/7 activity assay by DNA fluorescent dye containing caspase 3/7 cleavage site. Values are expressed as the mean ± SD from three independent experiments. * *p* < 0.05, ** *p* < 0.01, *** *p* < 0.001.

We next examined whether Manz A induced apoptosis in HCT116 cells. FITC-conjugated annexin V and PI double stained assay by flow cytometry was conducted. We found that Manz A significantly increased the early apoptotic cell population (annexin V positive cells) from 2% to 16% (Figure 4A). Mitochondrial membrane potential (MMP) is an important indicator for mitochondrial integrity and widely used to study apoptosis. Here, we used JC-1 staining and flow cytometry to detect the MMP. Tetraethylbenzimi-dazolylcarbocyanine iodide (JC-1), a lipophilic cationic dye, enters and accumulates in the electronegative interior of mitochondria where the probe aggregated and its fluorescent property changes. The dye forms complexes known as aggregates that yield a red fluorescence in healthy cells. During the early stage of programmed cell death, mitochondrial membrane potential decreases due to the opening of the mitochondrial permeability transition pores. This mitochondrial disruption results in the release of cytochrome c into the cytosol, which in turn triggers the downstream apoptotic cascades. Due to the loss of MMP, JC-1 remains as monomer which exhibits green fluorescence. In the present results, we showed that Manz A significantly reduced the fluorescence ratio of red to green, indicating that depolarization of MMP occurred during apoptosis (Figure 4B).

We then examined whether caspase cascade was triggered in the Manz A induced apoptosis. We applied a DNA fluorescent dye containing caspase 3/7 cleaved sequence DEVD to specify the activation of caspase-3/7 in apoptotic cells. We demonstrated that Manz A treatment caused a significant increase in fluorescent positive cells, showing that caspase-3/7 was activated by Manz A (Figure 4C). Collectively, our findings suggest that Manz A induce apoptotic cells death through mitochondria- and caspase-dependent pathways in HCT116 cells.

### 2.5. Epithelial–Mesenchymal Transition (EMT) Is Inactivated in Manz A Treated HCT116 Cells

**Figure 5:**
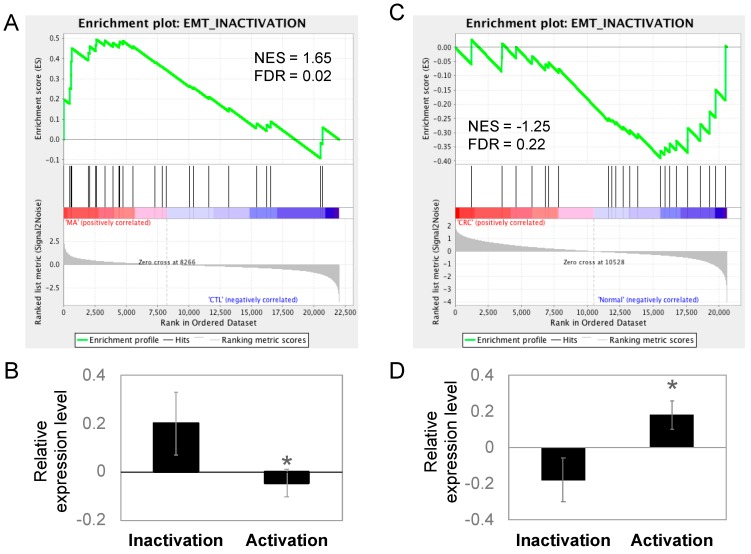
EMT was inactivated in Manz A treated HCT116 cells and normal colorectal tissues. Genes involved in EMT inactivation and activation were collected as two gene sets for GSEA to test the gene expression pattern between (**A**) MA-treated and DMSO-treated HCT116 cells or (**C**) the pattern between CRC and healthy control clinical tissues. Relative gene expression was ranked in descending order and colored from red to blue in response to MA (**A**) or occurrence of disease (**C**). Green line indicates the profile of running ES score and black lines represent the positions of gene set members on the rank ordered list according to their relative levels in Manz A (MA) compared to DMSO vehicle control (CTL) or CRC tumors (CRC) compared to biopsies from healthy controls (Normal). Relative expression levels of genes involved in EMT inactivation and activation were compared in (**B**) MA-treated HCT116 and (**D**) clinical tissues. Data is shown in mean expression of Z-transformed expression level. * *p* < 0.05.

The migratory capacity of cancer cells is necessary for metastasis and the acquisition of metastatic capability is associated with EMT in cancer cells. During the process of EMT, epithelial cells lose cell-to-cell contacts and gain expression of mesenchymal factors, enabling migration and invasion into surrounding stroma to facilitate metastasis. Because epithelial-like changes in cell morphology was observed during Manz A treatment, we performed an enrichment analysis on EMT regulators to evaluate whether Manz A also altered the EMT at gene expression levels. We divided the EMT-related genes according to their function into two portions, the activating and inactivating genes. By applying GSEA, we revealed that genes involved in EMT inactivation was significantly enriched in Manz A treated condition (Figure 5A, FDR = 0.02) and genes for activating EMT was unchanged (FDR = 0.84). We subsequently compared the mean expression of genes involved in EMT regulation and found that genes inactivating EMT were significantly higher than those activating EMT (Figure 5B), implying that Manz A inhibited the EMT process through gene expression regulation and might cause the cells to lose their migratory ability. Additionally, we compared the expression of EMT regulating genes in clinical subjects. The genes inactivating EMT were enriched in healthy controls (Figure 5C) and in lower expression than genes activating EMT in CRC patients (Figure 5D), suggesting that CRC patients who exhibiting lower expression in EMT inactivating genes are potent responders to Manz A treatment due to the effect that Manz A activates these genes.

### 2.6. Manz A Suppresses EMT Markers and Migration of Colorectal Carcinoma Cells

**Figure 6:**
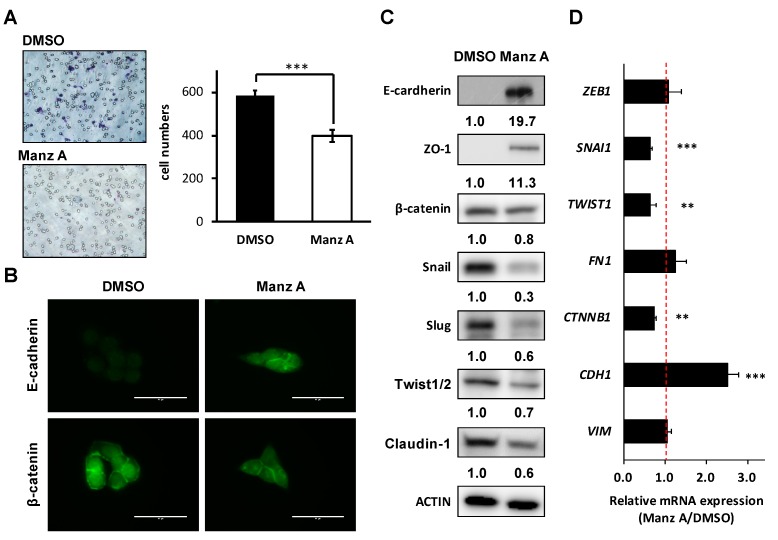
Manz A reversed epithelial-mesenchymal transition (EMT) and decreased cell mobility in HCT116 cells. (**A**) Transwell assay of HCT116 cells. Cells were pre-treated with 5 µM Manz A or DMSO for 24 h before seeded onto 24-well transwells. Migrated cell were stained and counted after 8 h; (**B**) Immunofluorescence of stained E-cadherin and β-catenin in HCT116 cells were monitored in the presence or absence of Manz A. Bars indicate 40 μm (**C**) Western blot analyses of the EMT regulator levels in response to Manz A treatment in HCT116 cells. ACTIN was used as an internal control. Relative expression level of each protein was densitometrically estimated and normalized to that of ACTIN under the same treatment; (**D**) Relative mRNA expression of EMT-related genes in HCT116 cells in response to Manz A treatment. The mRNA level of GAPDH was used as an internal control. ** *p* < 0.01, *** *p* < 0.001.

Loss of epithelial cadherin (E-cadherin) is considered to be a critical molecular feature of EMT. E-cadherin acts as a tumor suppressor which inhibits invasion and metastasis. Several signaling pathways including TGFβ, PI3K/AKT, and WNT signaling promote EMT by inhibiting glycogen synthase kinase-3β (GSK3β) to stabilize β-catenin, which translocates to the nucleus to regulate the transcription factors and promote a gene expression profile that favors EMT [23]. Therefore, we assessed the effects of Manz A on cell migration and EMT makers in HCT116 cells. We found that Manz A significantly reduced the migratory ability (Figure 6A). Subsequently, we revealed that Manz A increased E-cadherin expression and inhibited the nuclear translocation of β-catenin (Figure 6B). Moreover, Manz A induced the protein expression of several epithelial markers including E-cadherin and Zona occludens-1 (ZO-1) and reduced that of β-catenin, tight junction protein claudin-1, and EMT transcriptional regulators Snail, Slug, and Twist1/2 (Figure 6C). Simultaneously, CDH1 (encoding E-cadherin), SNAI1, TWIST1, and CTNNB1 (encoding β-catenin) were consistently regulated at the mRNA levels (Figure 6D). Collectively, these results demonstrate that Manz A reverses EMT and drives an epithelial-like phenotype, thereby suppressing the migration of colorectal carcinoma cells.

## 3. Discussion

Manz A was reported to exhibit anticancer activities in pancreatic cancer cells [13,20,21], yet the role of Manz A in CRC remains poorly understood. Consistent with prior studies, we demonstrated that Manz A significantly inhibited the proliferation of colorectal carcinoma cells (Figure 1). To comprehensively understand the molecular mechanism induced by Manz A, we obtained gene expression profile using microarray analysis and implemented a series of bioinformatics analyses. Using functional enrichment analysis on the transcriptome data, we revealed that Manz A caused downregulation in several fundamental pathways including cell cycle, DNA repair, and mRNA metabolism, and triggered apoptosis to inhibit the cell survival of CRC cells (Figure 2). Manz A caused cell cycle arrest and apoptosis was subsequently confirmed (Figure 3 and Figure 4). Based on the results from GSEA, we found that genes involved in EMT inactivation was significantly enriched in Manz A treated condition, explaining the epithelial-like phenotype we observed in cells treated with Manz A (Figure 5). We further validated the results by assessing the expression of EMT markers at both transcriptional and translational levels and showed the evidence of EMT reversal at molecular level which lead to diminishment of cell migration (Figure 6).

In mammalian cells, G_1_–S transcription depends on the transcription factor E2F family and their heterodimerization partner proteins during cell cycle process. Dysregulation of E2F function is frequently observed in cancer. In addition to E2F proteins, phosphorylation of their pocket proteins such as RB by cyclin D–CDK4/6 induces the transcription of G_1_–S target genes, such as the gene encoding cyclin E. Then cyclin E–CDK2 phosphorylates their pocket proteins, thus generating a positive feed ward loop to ensure the progression of cell cycle [24,25]. In the present study, we performed transcriptional activity analysis and found that E2F was downregulated by Manz A treatment (Figure S2). We further verified that Manz A induced cell cycle arrest at G_0_/G_1_ phase and downregulated the expression of CDK2, CDK4 and cyclin D1 through p53/p21 and p27 pathway (Figure 3).

Here, we also found that CREB, ATF and AP-1 were enriched in transcriptional activity analysis under Manz A treatment (Figure S2). AP-1 transcription factor can exert both oncogenic and tumor suppressive effects by regulating genes involved in cell proliferation, apoptosis, angiogenesis, and tumor invasion. Forming a dimeric complex, AP-1 interacts with JUN, FOS, ATF, or MAF (musculoaponeurotic fibrosarcoma) protein families. JUNB and JUND can repress crucial regulators of cell-cycle progression including gene encoding cyclin D1 [26]. Accumulating evidence indicates that increased AP-1 activity leads to apoptosis. c-Jun and c-Fos regulate the gene encoding Fas ligand, which triggers apoptosis [27], suggesting that Manz A not only activate mitochondria-mediated apoptosis (Figure 4) but also trigger an extrinsic apoptotic pathway through TRAIL activation as previously reported [20]. Inactivation of JunB in myeloid cells leads to reduced apoptosis with increased expression of the anti-apoptotic Bcl2 and Bcl-xl genes [28]. The activating transcription factor/cyclic AMP response element binding (ATF/CREB) family is involved in various cellular processes including cell stress responses, cell survival, and cell growth in cancer. Similar to AP-1, the ATF/CREB family also play roles as both a tumor suppressor and oncogene. ATF3, an adaptive-response gene, exhibits tumor suppressor function in the development of human colorectal cancer [29]. ATF3 can enhance the activation of p53 [30] and down-regulate cyclin D1 [31]. ATF3/CREB activation induces apoptosis in HCT116 human colorectal cancer cells [32,33]. Collectively, our results indicate that Manz A might induce apoptosis by upregulating transcriptional activities of AP-1 and ATF (Figure 2, Figure 4 and Figure S2). Nevertheless, the precise role of Manz-A-upregulated ATF and CREB in human colorectal cancer needs to be investigated further in the future.

In CRC patients, metastases are the main cause of cancer-related mortality. Approximately half of CRC patients will develop liver or lung metastasis during disease progression [34,35,36]. By comparing the expression pattern of EMT regulators in CRC patients and healthy controls, we revealed that inactivation of EMT in healthy control subjects is significantly enriched and might be critical to prevent the disease occurrence (Figure 5C,D). EMT has been considered to be a fundamental event in cancer metastasis. We showed that Manz A markedly induced E-cadherin expression with surface-surrounding localization, decreased EMT transcriptional regulators such as Snail, Slug, and Twist1/2, and prevented the nuclear translocation of β-catenin. Furthermore, a decreased expression of Claudin-1 was observed. The claudins are a family of integral membrane proteins forming the tight junction to maintain the barrier function that exists between epithelial cells. Claudin-1 is one of the most dysregulated claudins in human cancers and functions as a cancer promoter and tumor suppressor depending on cancer type [37]. Claudin-1 has been reported to overexpress in colon cancer, in particular in metastatic cells with mislocalization from the cell membrane to the cell nucleus and cytoplasm, and regulate cellular transformation and metastasis in xenograft tumor model through its effects on E-cadherin and β-catenin/Tcf signaling [38]. As a result, we hypothesized that Manz A might disrupted the EMT upstream signaling through preventing nuclear localization of β-catenin to diminish the activity of EMT transcription factors and reestablish the tight junction and adherent junction between epithelial cells. Based on the effects on activation of genes inactivating EMT, we assumed that CRC patients with tumors appearing in poor histological differentiation are potentially benefit from Manz A treatment. Tumors with dedifferentiated phenotypes are known to have the acquired capability for metastasis through the induction of epithelial–mesenchymal transition (EMT) [39]. Our results suggest that Manz A could be expected to have a potential role in a personalized selectivity in CRC treatment, for patients experienced recurrence and distal metastasis.

## 4. Materials and Methods

### 4.1. Reagents and Chemicals

Manzamine A (Manz A), with a purity of more than 98%, was obtained from Enzo Life Sciences (Farmingdale, NY, USA). The stock solution of Manz A was prepared at a concentration of 10 mM in dimethyl sulfoxide (DMSO, Sigma-Aldrich, St. Louis, MO, USA) and stored at −20 °C. Manz A was diluted in culture medium to obtain the desired concentration. DNase-free RNase A, propidium iodide (PI), and Triton X-100 were purchased from Sigma-Aldrich. Annexin V: FITC Apoptosis Detection Kit I was purchased from BD Biosciences (San Diego, CA, USA). 0.05% trypsin-EDTA (1X) were purchased from Caisson Labs (Smithfield, UT, USA). Trypan blue, M-PER™ Mammalian Protein Extraction Reagent, Halt™ Protease and Phosphatase Inhibitor Cocktail, and Electrochemiluminescence (ECL) HRP substrate were purchased from Thermo Fisher Scientific (Boston, MA, USA). CellTiter 96^®^ AQueous One Solution Cell Proliferation (MTS) assay was purchased from Promega (Madison, WI, USA). Bicinchoninic acid (BCA) assay kit was purchased from T-Pro Biotechnology (New Taipei County, Taiwan). Antibodies against E-cadherin, β-catenin, Snail, Slug, Claudin-1, and ZO-1 were purchased from Cell Signaling Technology (Beverley, MA, USA). Antibodies against Cyclin D1, p21^cip1^, p27^kip1^, and Twist1/2 were purchased from GeneTex (Irvine, CA, USA). Antibodies against p53 and ACTIN were purchased from EMD Millipore (Billerica, MA, USA). Antibodies against CDK2 and CDK4, and the goat anti-rabbit/mouse antibody IgG were purchased from Abcam (Cambridge, UK). TRI Reagent was purchased from Invitrogen (Carlsbad, CA, USA). TURBO DNA-free™ Kit was purchased from Ambion (Austin, TX, USA). Low Input Quick-Amp Labeling kit and CyDye Cy3 were purchased from Agilent Technologies (Santa Clara, CA, USA).

### 4.2. Cell Culture

Human colorectal carcinoma cell lines HCT116, HT-29, and DLD-1 were obtained from the American Type Culture Collection (ATCC, Manassas, VA, USA). All cell lines were cultured in RPMI 1640 supplemented with 10% fetal bovine serum in a humidified atmosphere containing 95% air and 5% CO_2_ at 37 °C. 

### 4.3. Cell Proliferation Analysis and Colony Formation Assay

The effects of Manz A on cell proliferation were assessed by using the MTS assay (CellTiter 96 Aqueous One Solution cell proliferation assay; Promega, Madison, WI, USA). Briefly, HCT116, DLD-1, and HT-29 cells were seeded onto 96-well plates at a density of 7000 cells/well with 100 μL of complete medium/well for 24 h prior to Manz A treatment. MTS reagent was added to each well at the indicated incubation times and incubated at 37 °C for 90 min in the dark. The spectrophotometric absorbance of colored formazan dye generated by viable cells was measured on an Epoch Microplate Spectrophotometer (BioTek, Winooski, VT, USA) at 490 nm. The relative cell viability is defined as the ratio of the absorbance at 490 nm of Manz A treated cells to that of DMSO treated ones. All assays were performed in at least three independent experiments.

To evaluate the long-term inhibitory effects of Manz A on cell proliferation, a colony formation assay was performed as described previously [40] with modifications. HCT116 cells were seeded onto 6-well plates at a density of 2.5 × 10^5^ cells/well. After attachment, cells were treated with 5 μM Manz A for 24 h. The cells were then detached, reseeded onto 6-well plates at a density of 1000 cells/well, and cultured with drug-free medium. Cells were cultured for 7 days to allow the colonies to form. Culture medium was changed every two days to ensure the supplement of nutrients. Formed colonies were fixed in methanol, stained with 1:10 Giemsa stain (Sigma-Aldrich), photographed under a microscope and counted.

### 4.4. Microarray Analysis

Total RNA was isolated using the TRI Reagent according to the manufacturer’s instructions and treated with the TURBO DNA-free™ Kit to remove genomic DNA contamination. 0.2 μg of total RNA was amplified by a Low Input Quick-Amp Labeling kit and labeled with Cy3 during the in vitro transcription process. 0.6 μg of Cy3-labled cRNA was fragmented to an average size of about 50–100 nucleotides by incubation with fragmentation buffer at 60 °C for 30 mins. Correspondingly fragmented labeled cRNA is then pooled and hybridized to Agilent SurePrint G3 Human GE 8 × 60 K Microarray (Agilent Technologies) at 65 °C for 17 h. After washing and drying by nitrogen gun blowing, microarrays are scanned with an Agilent microarray scanner (Agilent Technologies) at 535 nm for Cy3. Scanned images are analyzed by an image analysis and normalization software Feature extraction 10.5.1.1 software (Agilent Technologies) to quantify signal and background intensity for each feature.

### 4.5. Functional Enrichment Analysis and Data Visualization

To interpret the results from the microarray analysis, we applied functional enrichment analysis in two ways, over-representation analysis (ORA) and functional class sorting (FCS). For ORA, significantly regulated genes (*p* < 0.05) with more than 2-fold changes were functionally enriched using DAVID (https://david.ncifcrf.gov) [41]. Data from Reactome pathway database [42] was used for interpreting the involved pathways. In parallel, we utilized gene set enrichment analysis (GSEA, http://software.broadinstitute.org/gsea/index.jsp) [43] for FCS according to online documentation. In brief, raw signal data from microarray was used as the input expression file. The genes were ranked according to the expression difference between MA-treated and DMSO control cells using the signal to noise parameter. 615 gene lists from C3 (TFT: transcription factor targets) in MSigDB was assign as the gene set. A non-parametric running sum statistic termed the enrichment score (ES) was measured to obtain the association between gene set and expression ranking. Permutation testing was applied to assess the statistical significance of the maximum ES, which is calculated as the fraction of the 1000 random permutations on the gene set. The unadjusted nominal p value estimates the statistical significance of a gene set and the false discovery rate (FDR) statistic adjusting for gene set size and multiple hypothesis testing were reported. A FDR of 0.25 was selected as a threshold as recommended. To visualize the functional enrichment results, a Cytoscape [44] plugin Enrichment Map [45] was applied using default setting of *p*-value cutoff = 0.005, FDR cutoff = 0.1, and overlap coefficient = 0.5. For tissue expression data (GSE9348 [46]), the genes were ranked according to the expression difference between CRC patients and healthy controls and the enrichment analysis in EMT genes were analyzed by GSEA with the same parameters for Manz A dataset.

### 4.6. Flow Cytometry Analysis

HCT116 cells were seeded onto 6-well plates at a density of 2.5 × 10^5^/well for 24 h and treated with 5 μM Manz A for 24 h. Cells were then washed with PBS and harvested with trypsin-EDTA solution. For the cell cycle analysis, cells were fixed with 70% ethanol at −20 °C overnight, washed with ice-cold PBS, incubated with 0.1 mg/mL DNAse-free RNAse A, and stained with 50 mg/mL PI. DNA content of PI-stained single-cell suspensions was acquired with a FACSCalibur flow cytometer equipped with CellQuest software (BD Biosciences, San Jose, CA, USA). For the apoptosis assay, Annexin V: FITC Apoptosis Detection Kit I was used. The cells were suspended with 100 μL of binding buffer (10 mM HEPES/NaOH, 140 mM NaCl, 2.5 mM CaCl2, pH 7.4) and stained with 2 μL of FITC-conjugated Annexin V and 2 μL of PI (50 μg/mL) for 15 mins at room temperature in the dark and analyzed with a FACSCalibur flow cytometer equipped with CellQuest software (BD Biosciences).

For flow cytometric detection of mitochondrial membrane potential (MMP, Δψm), cells were washed with PBS once and incubated with 2 μM JC-1 in 5% CO_2_ incubator at 37 °C for 30 min after being treated with 5 μM Manz A for 24 h. Then the stained cells were harvested with trypsin-EDTA solution into plastic tubes fitted with the flow cytometer and centrifuged at 1200 rpm for 5 min to remove the dye and resuspended with PBS buffer. The MMP of Manz A treated cells was quantified by FACSCalibur flow cytometer equipped with CellQuest software (BD Biosciences) using 488 nm excitation. Cells with red JC-1 aggregates and apoptotic cells with collapsed mitochondria containing green JC-1 monomers were detected in FL2 and FL1 channels, respectively.

### 4.7. Caspases 3/7 Activities Assay

Detection of caspases 3 and 7 activation was performed by using the CellEvent™ Caspase 3/7 Green Detection Reagent which is a four-amino acid peptide (DEVD) conjugated to a nucleic acid-binding dye. Upon the activation of caspase-3/7 in apoptotic cells, the DEVD peptide is cleaved and the dye is released from the peptide to bind DNA and generate a bright green fluorescence in nucleus. 10^5^ HCT116 cells were seeded onto 12-well plates for 24 h. After attachment, cells were treated with DMSO control or 5 μM Manz A for 24 h and then washed with PBS once. CellEvent™ Caspase 3/7 Green Detection Reagent was added to the wells at the final concentration of 5 μM. After incubation for 30 min at 37 °C in the dark, cells were then imaged by a EVOS^®^ FL Auto Cell Imaging System (Thermo Fisher Scientific) with a FITC filter.

### 4.8. Transwell Migration Assay

Migratory capacity of colorectal carcinoma cells was assayed using modified Boyden chamber assay with transwell consisting of a polycarbonate membrane at the bottom with 8 μm pore (Corning Inc., Acton, MA, USA). HCT116 cells were seeded overnight and treated with DMSO control or 5 μM Manz A for 24 h. Cells were then washed and harvested. The cells suspension was diluted to an optimal density and loaded into the transwell placed in wells of 24-well plates without touching the membrane or introducing air bubbles. The lower chamber contained Manz A-free RPMI medium supplied by 10% FBS as a chemoattractant. Following incubation for 8 h, cells in the upper chamber were scraped off and the membranes were fixed in methanol for 20 min. Migrated cells remaining on the bottom surface of the membrane were counted after stained with 0.1% crystal violet (Sigma-Aldrich) for 10 min.

### 4.9. Western Blot Analysis

HCT116 cells treated with DMSO or 5 μM Manz A for 24 h were lysed for 30 min in ice-cold lysis buffer containing protease and phosphatase inhibitor cocktail. The protein concentration was determined by Pierce bicinchoninic acid (BCA) protein assay kit (Thermo Scientific). Thirty μg protein was subjected to SDS-PAGE, resolved on a 7.5–15% polyacrylamide gel, and transferred onto a polyvinylidene difluoride (PVDF) membrane. The membrane was then blocked in 5% bovine serum albumin (BSA) for 1 h and incubated with the appropriate primary antibody overnight at 4 °C. After three washes with Tris-buffered saline containing 0.05% Tween-20 (TBST), the membrane was incubated with secondary anti-rabbit or anti-mouse IgG antibodies (1:15,000) for 1 h at room temperature. The immunoreaction was visualized using the ECL HRP substrate and detected using a Luminescent Image Analyzer Amersham Imager 600 (GE Healthcare Life Sciences, MA, USA). The band intensity was quantified using ImageJ software.

### 4.10. RNA Isolation, Reverse Transcription and Quantitative Real-Time PCR (qPCR) Analysis

Total RNA from DMSO- or Manz-A-treated HCT116 cells was extracted using TRIzol™ Reagent (Invitrogen) followed by purification with Direct-zol RNA MiniPrep (Thermo Scientific). cDNA was reverse transcribed from 2 µg total RNA using RevertAid RT Reverse Transcription Kit (Thermo Scientific). mRNA level of genes of interests was measured with gene-specific primers and Power SYBR Green Master Mix (Thermo Fisher Scientific). In brief, 1 ng of cDNA was added to the mix containing appropriate primer sets (400 nM) and SYBR green in a 10 μL reaction volume. All samples were analyzed in triplicate. Real-time PCR analyses were performed with a Applied Biosystems StepOnePlus™ Real-Time PCR System (Thermo Fisher Scientific). Amplification of all genes was performed under the following cycling conditions: denaturation at 95 °C for 10 mins followed by 40 cycles for 15 s at 95 °C and 30 s at 60 °C. Synthesis of DNA product of the expected size was confirmed by melting curve analysis and DNA electrophoresis. Relative quantification was done by ΔΔCt method as fold change normalized to internal control GAPDH and vehicle control. Primers used for qPCR analysis are listed in Table 1.

### 4.11. Immunocytochemistry

HCT116 cells were cultured on sterile glass coverslips and treated with 5 μM of Manz A for 24 h. Cells were fixed with 4% paraformaldehyde at room temperature for 15 min followed by permeablized with 0.025% Triton X-100/PBS for 10 min. Cells were then blocked in blocking solution consisting of 5% (*w*/*v*) BSA in TBST at room temperature for 1 h and incubated with primary antibodies overnight at 4 °C. After being washed three times with PBS containing 0.1% Tween-20 (PBST), cells were further incubated with secondary antibodies (Alexa Fluor^®^ 488 dye, LifeTechnologies, Gaithersburg, MD, USA) for 1 h at room temperature in the dark. After three times of PBST washes, cells were mounted with ProLong^®^ Gold Antifade Mountant with 4′,6-diamidino-2-phenylindole (DAPI). Fluorescent images were taken using an EVOS^®^ microscope (Thermo Fisher Scientific).

### 4.12. Statistical Analysis

The data was shown as the means ± standard deviations (SD). A two-tailed Student’s *t*-test was used to determine the significance of differences between DMSO control- and Manz-A-treated groups. Statistical significance was reached when the *p*-value less than 0.05. Experiments were repeated independently at least three times (*n* = 3).

## 5. Conclusions

In summary, we demonstrated that Manz A exhibits an antiproliferative effect on human colorectal carcinoma cells and displays broad effects on gene expression to downregulate fundamental maintenances of cell survival and induce apoptotic cell death and EMT inactivation. We validated that Manz A causes cell cycle arrest at G_0_/G_1_ phase through the activation of p53, p21, and p27 CKIs and triggers caspase-dependent apoptosis via mitochondrial membrane potential depletion. Specifically, we found that the EMT and migratory ability are inhibited, suggesting Manz A can serve as a potential anticancer drug for CRC patients baring tumors undergoing EMT process and developing distal metastasis.

## Figures and Tables

**Table 1:** Sequences of qPCR primers.

Gene	Forward (5′ to 3′)	Reverse (5′ to 3′)
*VIM*	AGTCCACTGAGTACCGGAGAC	CATTTCACGCATCTGGCGTTC
*CDH1*	ATTTTTCCCTCGACACCCGAT	TCCCAGGCGTAGACCAAGA
*CTNNB1*	GTCTGAGGACAAGCCACAAGA	TCCCTGGGCACCAATATCAAG
*FN1*	GGCCAGTCCTACAACCAGTAT	TCGGGAATCTTCTCTGTCAGC
*TWIST1*	GCTGAGCAAGATTCAGACCCT	TCCATCCTCCAGACCGAGAA
*SNAI1*	AAGGGACTGTGAGTAATGGCTG	TAGTTCTGGGAGACACATCGGT
*ZEB1*	CAGCTTGATACCTGTGAATGGG	TATCTGTGGTCGTGTGGGACT

## Supplementary Materials

The following are available online at http://www.mdpi.com/1660-3397/16/8/252/s1, Figure S1: over-representation analysis of differentially expressed genes obtained from microarray analysis, Figure S2: Enrichment map of functional enrichment in transcription factor targets from GSEA result.
