# Supplementary material for: Manzamine A Exerts Anticancer Activity against Human Colorectal Cancer Cells
Source: Mar Drugs. 2018 Jul 29;16(8):252. doi: 10.3390/md16080252 (PMC6117705; doi:10.3390/md16080252)
Supplement: Supplementary file 1 [file marinedrugs-16-00252-s001.pdf]

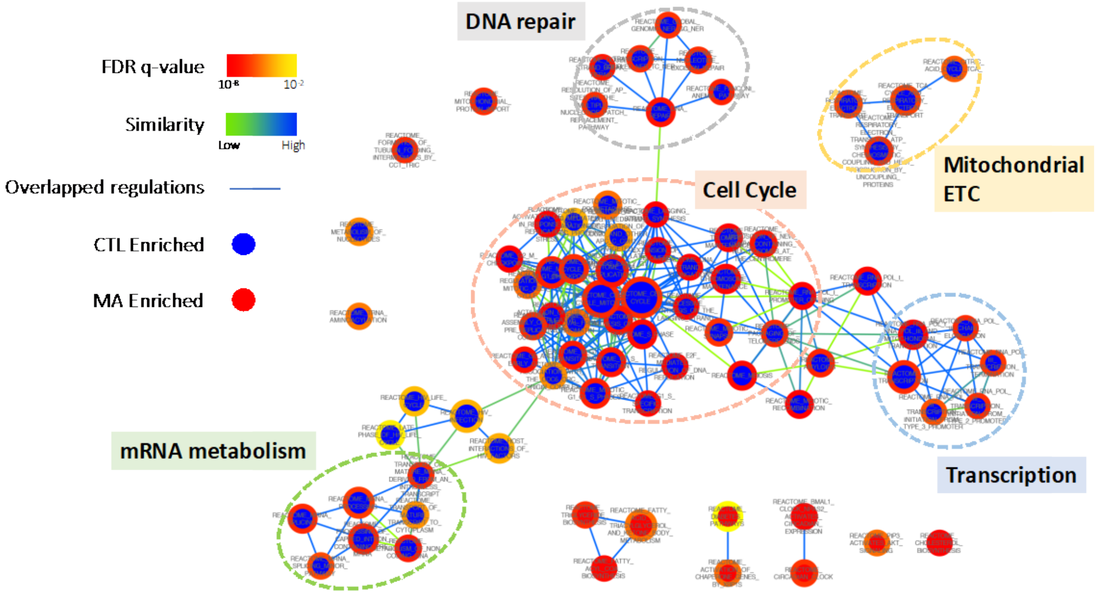

Figure S1. Enrichment map of functional enrichment in REACTOME of differentially expressed (DE) genes. DE genes from microarray analysis were subjected to DAVID for enriching pathway terms in REACTOME. Enrichment results were visualized in Cytoscape 3.0 using Enrichment Map plugin. Each node indicates an enriched term and edges represent overlapped genes between terms. The color of node boarder and edge were shown according to enrichment FDR and similarity, respectively. The color of nodes in blue and red refer as enriched functions in CTL and Manz A treatment.

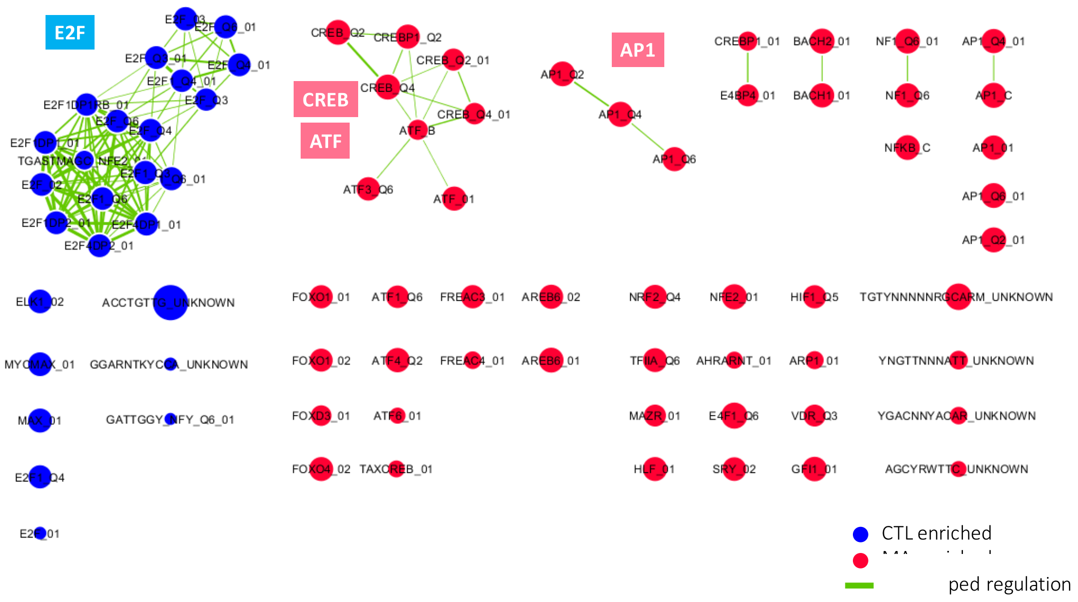

Figure S2. Enrichment map of functional enrichment in transcription factor targets obtained from GSEA result. GSEA was performed on microarray data for enriching transcription factors. Enrichment results were visualized in Cytoscape 3.0 using Enrichment Map plugin. Each node indicates an enriched term and edges represent overlapped genes between terms. The color of nodes in blue and red refer as enriched functions in CTL and Manz A treatment. The size of node and edge width represent the number of genes enriched in each term and overlapped between terms, respectively.
